# Laparoscopic Distal Pancreatectomy Following Prior Upper Abdominal Surgery (Pancreatectomy and Prior Surgery)

**DOI:** 10.1007/s11605-020-04858-2

**Published:** 2020-11-10

**Authors:** Mushegh A. Sahakyan, Tore Tholfsen, Dyre Kleive, Sheraz Yaqub, Airazat M. Kazaryan, Trond Buanes, Bård Ingvald Røsok, Knut Jørgen Labori, Bjørn Edwin

**Affiliations:** 1 The Intervention Center, Oslo University Hospital, Pikshospitalet, 0027 Oslo, Norway; 2 Department of Surgery N1, Yerevan State Medical University After M. Heratsi, Yerevan, Armenia; 3 Department of Research & Development, Division of Emergencies and Critical Care , Oslo University Hospital , Oslo, Norway; 4 Department of HPB Surgery, Oslo University Hospital, Pikshospitalet, Oslo, Norway; 5 Department of Gastrointestinal Surgery, Østfold Hospital Trust, Grålum, Norway; 6 Department of Faculty Surgery N2, I.M. Sechenov First Moscow State Medical University, Moscow, Russia; 7 Institute of Clinical Medicine, Medical Faculty, University of Oslo, Oslo, Norway

**Keywords:** Laparoscopy, Pancreatectomy, Surgery, Abdomen, Morbidity

## Abstract

**Background and Purpose:**

Previous abdominal surgery can be a risk factor for perioperative complications in patients undergoing laparoscopic procedures. Today, distal pancreatectomy is increasingly performed laparoscopically. This study investigates the consequences of prior upper abdominal surgery (PUAS) for laparoscopic distal pancreatectomy (LDP).

**Methods:**

Patients who had undergone LDP from April 1997 to January 2020 were included. Based on the history and type of PUAS, these were categorized into three groups: minimally invasive (I), open (II), and no PUAS (III). To reduce possible confounding factors, the groups were matched in 1:2:4 fashion based on age, sex, body mass index (BMI) and American Society of Anesthesiology grade.

**Results:**

After matching, 30, 60, and 120 patients were included in the minimally invasive, open and no PUAS groups, respectively. No statistically significant differences were found in terms of intraoperative outcomes. Postoperative morbidity, mortality and length of hospital stay were similar. Open PUAS was associated with higher Comprehensive Complication Index (33.7 vs 20.9 vs 26.2, *p* = 0.03) and greater proportion of patients with ≥ 2 complications (16.7 vs 0 vs 6.7%, *p* = 0.02) compared with minimally invasive and no PUAS. Male sex, overweight (BMI 25–29.9 kg/m^2^), diagnosis of neuroendocrine neoplasia, and open PUAS were risk factors for severe morbidity in the univariable analysis. Only open PUAS was statistically significant in the multivariable model.

**Conclusions:**

PUAS does not impair the feasibility and safety of LDP as its perioperative outcomes are largely comparable to those in patients without PUAS. However, open PUAS increases the burden and severity of postoperative complications.

## Introduction

Laparoscopic distal pancreatectomy (LDP) is becoming a standard modality in the treatment of lesions in the pancreatic body and tail.^1^,^2^ Together with an expansion of selection criteria, disease-, and patient-specific parameters are gaining more attention in the decision-making process. The association between age, obesity, functional status, tumor size, stage and the outcomes of LDP has been addressed in the literature, also by our research group.^3^–^8^ Another important aspect in the planning and execution of LDP is patient’s previous surgical history, particularly, the presence of prior upper abdominal surgery (PUAS).

Intraperitoneal adhesions following PUAS have been reported in up to 90% of patients.^9^ These may potentially increase the technical difficulty of laparoscopic procedures resulting in visceral injury, bleeding, and conversion. In laparoscopic hepato-pancreato-biliary (HPB) surgery, PUAS has been associated with prolonged operative time, conversion, and postoperative morbidity.^9^–^13^ To the best of our knowledge, no studies examining the influence of PUAS on the outcomes of LDP have been published to date. Given the increasing number of candidates for this procedure, rigorous evaluation of periprocedural risks associated with PUAS is needed.

The aim of this report was to assess the consequences of PUAS for perioperative results of LDP. Thereby, the experience with LDP in a high-volume referral center for pancreatic surgery was analyzed.

## Material and Methods

Patients were operated at Oslo University Hospital, Rikshospitalet, between April 1997 and January 2020. Information on patient demographics, PUAS, comorbidities, clinical characteristics, perioperative outcomes, and complications was obtained from a prospectively maintained database. Patients without information of previous abdominal surgery were excluded. Based on the presence of PUAS and its technique the patients were enrolled in one of the following groups: minimally invasive (I), open (II), and no PUAS (III). These were compared in terms of perioperative outcomes. To reduce possible confounding factors, the minimally invasive, open and no PUAS groups were matched in 1:2:4 fashion based on the following covariates: age, sex, body mass index (BMI), and American Society of Anesthesiology (ASA) grade. Patients undergoing multiorgan resections or concomitant abdominal surgery were excluded for further standardization of the study groups. The study was approved by the hospital review board according to the guidelines provided by the regional ethics committee.

For more than 20 years LDP has been the standard procedure for lesions in the body and tail of the pancreas at our institution. Surgical technique and perioperative management of these patients have been described previously.^14^,^15^ LDP was performed by either senior consultant or staff surgeon. Open distal pancreatectomy was done in a very small proportion (< 4%) of selected patients—usually, due to the need for complex vascular reconstruction*.*

PUAS was defined as a surgical procedure performed in the upper portion of the peritoneal cavity, i.e., involving any organ located higher than the umbilicus. In open PUAS, a distinct laparotomy scar above the umbilicus was present. In minimally invasive PUAS, involvement of the upper portion of the peritoneal cavity was the key determinant regardless of where the surgical ports were placed. Patients with a history of both minimally invasive and open PUAS were added to the open group. PUAS included surgical procedures on the liver, gallbladder, bile ducts, pancreas, stomach, spleen, small intestine, kidney, adrenal glands, upper retroperitoneum, and diaphragm, as well as colectomy involving the upper abdomen in the dissection area. Conversely, colorectal resections excluding this area, lower abdominal surgery, and gynecological procedures were not considered as PUAS.

Based on the extent of PUAS, it was divided into two types—major and minor. Procedures involving one abdominal quadrant (e.g., cholecystectomy, splenectomy, nephrectomy, adrenalectomy) were considered as minor.^16^,^17^ Conversely, those involving > 1 abdominal quadrant were regarded as major. The latter included HPB surgery, procedures on the stomach, small bowel, and colon. Patients with a history of both minor and major PUAS were added to the major PUAS group.

Obesity classes were defined based on BMI and categorized according to the World Health Organization criteria, i.e., normal weight (BMI = 18.5–24.9 kg/m^2^), overweight (BMI = 25–29.9 kg/m^2^) and obese (BMI ≥ 30 kg/m^2^). Conversion to open surgery was defined as laparotomy at any time during surgery, not specifically related to the extraction of the specimen. Intraoperative unfavorable events were defined and graded according to the Oslo classification based on Satava approach to surgical error evaluation.^8^,^18^,^19^ Postoperative morbidity was defined as suggested by Clavien and Dindo.^20^ Grade IIIa–V complications were considered severe. The Comprehensive Complication Index (CCI) was used for comprehensive and accurate measurement of postoperative complications.^21^ Postoperative pancreatic fistula (POPF) was reported as suggested in the 2016 update from the International Study Group of Pancreatic Surgery (ISGPS).^22^ Postoperative hemorrhage was defined and graded according to the ISGPS.^23^ The 90 days from surgery definition was used for mortality and readmission.^24^

Mean (± standard deviation) and median (range) values are applied for normally and nonnormally distributed continuous data, respectively. Accordingly, the analysis of variance (ANOVA) and the Kruskal–Wallis test were used to compare normally and nonnormally distributed data, respectively. The post hoc test was used to verify statistically significant differences between the means, and the two-sided Mann–Whitney *U* test was used for the medians. The categorical variables were shown as numbers (percentages), and the chi-square test or Fisher’s exact test were used to compare these. The two-sided *p* value < 0.05 was considered statistically significant.

Univariable and multivariable analyses were run by using binary logistic regression model. Variables significant at the *p* value < 0.1 in the univariable analysis were added to the multivariable model, where the two-sided *p* value < 0.05 was considered statistically significant.

## Results

**Fig. 1:**
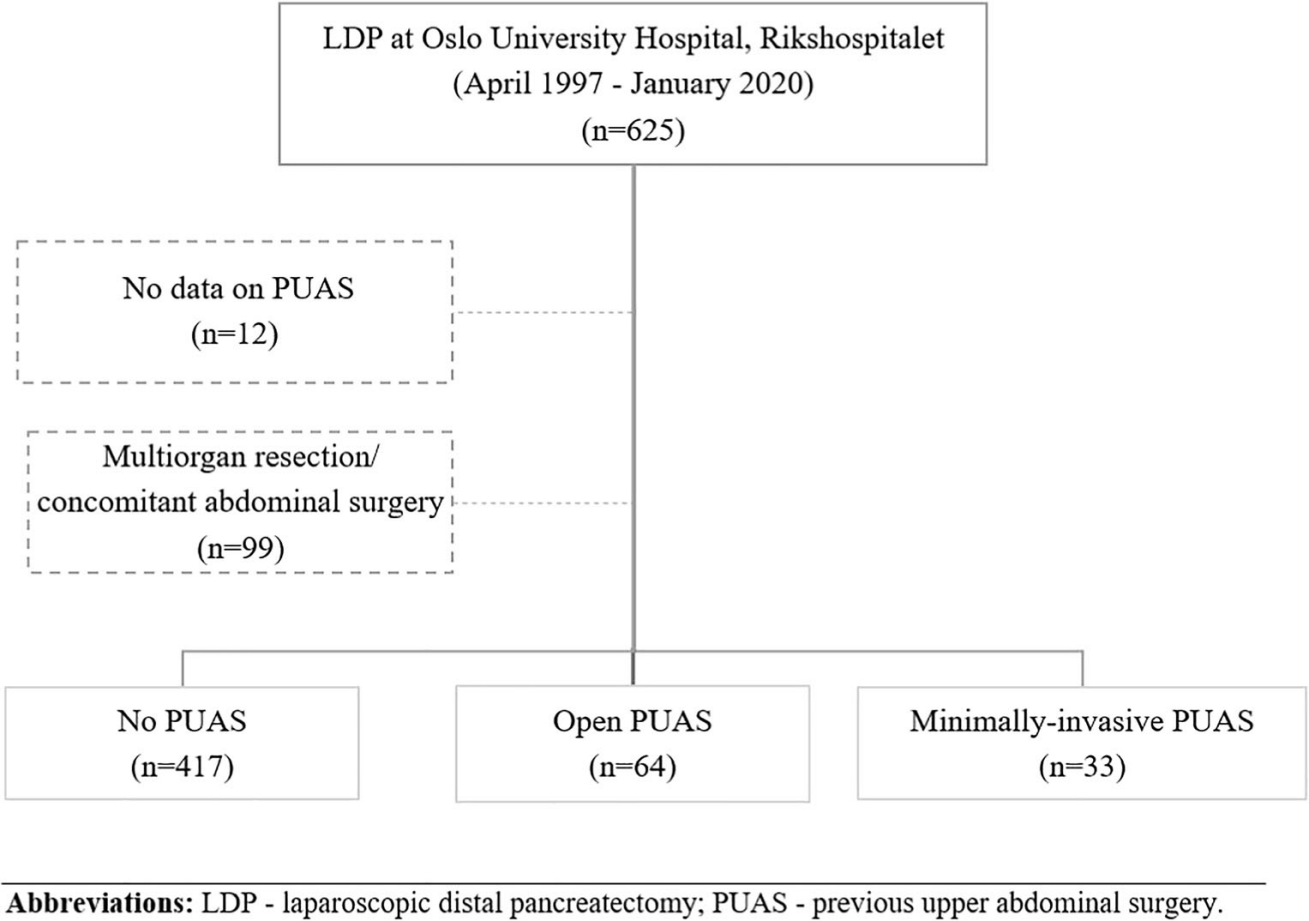
Study flow-chart. Abbreviations: LDP—laparoscopic distal pancreatectomy; PUAS—previous upper abdominal surgery

Six hundred twenty-five patients underwent LDP for all indications. Patients without information on PUAS (*n* = 12) and those with multiorgan resections/concomitant abdominal surgery (*n* = 99) were excluded (Fig. 1). As a result, 514 patients met the inclusion criteria. Of those, 417 (81.4%) had no surgical history, while 64 (12.1%) and 33 (6.4%) had open and minimally invasive PUAS, respectively. A total number of 78 procedures were performed in open PUAS group and 34 in the minimally invasive group (Table 1). The most common PUAS was cholecystectomy accounting for nearly a quarter of open and more than a half of minimally invasive procedures. There were 34 major open and 8 major minimally invasive PUAS.

**Table 1:** Description of previous upper abdominal surgical procedures in open and minimally invasive groups

Open	*n* (%)	Minimally invasive	*n* (%)
Cholecystectomy	19 (24.4%)	Cholecystectomy	20 (58.9%)
T-A* nephrectomy/kidney resection	15 (19.2%)	Hemi/subtotal/total colectomy	5 (14.8%)
Hemi/subtotal/total colectomy	10 (12.8%)	T-A* nephrectomy/kidney resection	3 (8.8%)
Surgery for small bowel obstruction	10 (12.8%)	T-A* ventral hernia repair	3 (8.8%)
Total/subtotal gastrectomy	8 (10.3%)	Total/subtotal gastrectomy	1 (2.9%)
Fundoplication/hiatal hernia repair	4 (5.1%)	Gastroenterostomy	1 (2.9%)
Liver resection	4 (5.1%)	Liver resection	1 (2.9%)
Splenectomy	3 (3.8%)		
Pancreatoduodenectomy	2 (2.6%)		
Gastroenterostomy	1 (1.3%)		
Bile duct resection	1 (1.3%)		
T-A* adrenalectomy	1 (1.3%)		
Total	78 (100%)	Total	34 (100%)

*Transabdominal

After matching, 30, 60, and 120 patients were included in the minimally invasive, open, and no PUAS groups, respectively. No statistically significant differences were found between the groups in terms of baseline characteristics, indications for surgery and spleen-preservation rates (Table 2). Proportions of major PUAS and staff surgeons performing LDP were similar between the groups. One patient without PUAS was converted to open approach due to massive intraoperative bleeding. Intraoperative parameters such as operative time, blood loss, blood transfusion, and intraoperative unfavorable incidents were comparable.

**Table 2:** Perioperative results of laparoscopic distal pancreatectomy in patients with and without previous upper abdominal surgery (matched cohort)

Parameters	Previous upper abdominal surgery			*p* value
	No (*n* = 120)	Open (*n* = 60)	M–I (*n* = 30)	
Age, years, mean (SD)	64 (12)	67 (11)	64 (11)	0.21
Body mass index, kg/m^2^, mean (SD)	26.5 (4.4)	25.4 (3.6)	26.8 (4.5)	0.17
Female sex, *n* (%)	64 (53.3%)	32 (53.3%)	16 (53.3%)	1.0
Comorbidities, *n* (%)	90 (75%)	48 (80%)	21 (70%)	0.56
Number of comorbidities, mean (SD)	1.5 (1.3)	1.4 (1.1)	1.5 (1.4)	0.82
Major PUAS, *n* (%)	-	26 (43.3%)	8 (26.7%)	0.12
ASA, median (range)	2 (2–3)	2 (2–3)	2 (2–3)	0.55
Diagnosis, *n* (%)				0.2
Ductal adenocarcinoma	20 (16.7%)	10 (16.7%)	3 (10%)	
Neuroendocrine neoplasm	37 (30.8%)	12 (20%)	4 (13.3%)	
Chronic pancreatitis	6 (5%)	2 (3.3%)	3 (10%)	
Other	57 (47.5%)	36 (60%)	20 (66.7%)	
Staff surgeon, *n* (%)	20 (16.7%)	7 (11.7%)	5 (16.7%)	0.66
Spleen-preserving procedure, *n* (%)	22 (18.3%)	7 (11.7%)	3 (10%)	0.45
Operative time, min, median (range)	158 (45–428)	155 (29–319)	142 (73–225)	0.22
Estimated blood loss, ml, median (range)	50 (0–2800)	50 (0–1900)	100 (0–800)	0.15
Conversion, *n* (%)	1 (0.8%)	0 (0%)	0 (0%)	1.0
Intraoperative unfavorable events, *n* (%)	7 (5.8%)	5 (8.3%)	2 (6.7%)	0.8
Grade II-III unfavorable events, *n* (%)	2 (1.7%)	1 (1.7%)	0 (0%)	1.0
Red blood cell transfusion, *n* (%)	7 (5.8%)	7 (11.7%)	0 (0%)	0.11
Postoperative complications, *n* (%)	42 (35%)	24 (40%)	9 (30%)	0.63
CCI, median (range) ^* ┼^	26.2 (8.7–54.2)	33.7 (8.7–100)	20.9 (8.7–26.2)	0.03
Cases with ≥ 2 complications, *n* (%) ^* ┼^	8 (6.7%)	10 (16.7%)	0 (0%)	0.02
Severe complications, *n* (%)	21 (17.5%)	18 (30%)	4 (13.3%)	0.07
Postoperative pancreatic fistula, *n* (%)	20 (16.7%)	13 (21.7%)	5 (16.7%)	0.7
Hemorrhage (grade B/C), *n* (%)	12 (10%)	5 (8.3%)	0 (0%)	0.19
Reoperation, *n* (%)	5 (4.2%)	6 (10%)	0 (0%)	0.13
Mortality, *n* (%)	0 (0%)	1 (1.7%)	0 (0%)	0.43
Readmission, *n* (%)	13 (10.8%)	8 (13.3%)	1 (3.3%)	0.35
Hospital stay, days, median (range)	6 (2–35)	5 (2–81)	5 (2–34)	0.39

*M–I* minimally invasive; *PUAS* previous upper abdominal surgery; *ASA* American Society of Anesthesiologists; *CCI* comprehensive complication index. *significant difference between no and open PUAS. ^┼^significant difference between open and minimally invasive PUAS

The rate of complications was similar, however the median CCI was higher in open PUAS compared with minimally invasive and no PUAS (33.7 vs 20.9 vs 26.2, *p* = 0.03). A significantly higher proportion of patients with ≥ 2 complications was observed in the open group compared with no PUAS (16.7 vs 6.7%, *p* = 0.04), while no such patients were found in the minimally invasive arm. Open PUAS showed a trend towards higher incidence of severe complications (30 vs 17.5 vs 13.3%, *p* = 0.07). Other postoperative outcomes were similar.

A univariable analysis was performed to identify risk factors for severe complications and to assess the potential role of PUAS (Table 3). Male sex, overweight, diagnosis of pancreatic neuroendocrine neoplasia, and open PUAS were associated with severe morbidity. In the multivariable analysis, only open PUAS correlated with severe morbidity increasing its likelihood more than three times compared with no PUAS–OR 3.42 (1.34–8.72), *p* = 0.01.

**Table 3:** Univariable and multivariable analyses of risk factors for severe morbidity following laparoscopic distal pancreatectomy

Variable	Univariate analysis		Multivariate analysis	
	OR (95% CI)	*p* value	OR (95% CI)	*p* value
Age	0.99 (0.96–1.02)	0.6		
Male sex	2.02 (1.02–4.0)	0.04	2.14 (0.84–5.44)	0.11
Obesity class (vs normal weight)				
Overweight	0.5 (0.23–1.12)	0.09	0.73 (0.28–1.86)	0.51
Obese	1.17 (0.5–2.73)	0.72		
Cardiovascular disease	1.4 (0.64–3.06)	0.4		
Hypertension	0.73 (0.36–1.51)	0.4		
COPD	1.37 (0.59–3.19)	0.46		
Diabetes mellitus	1.29 (0.54–3.12)	0.56		
Number of comorbidities	1.19 (0.93–1.53)	0.18		
ASA grades III–IV	1.76 (0.89–3.46)	0.1		
Diagnosis (vs other)				
Neuroendocrine neoplasm	1.99 (0.93–4.26)	0.08	2.24 (0.85–5.92)	0.11
Ductal adenocarcinoma	1.27 (0.49–3.3)	0.62		
Previous open UAS (vs none)				
Open	2.02 (0.98–4.17)	0.05	3.42 (1.34–8.72)	0.01
Minimally invasive	0.73 (0.23–2.29)	0.59		
Multiple (vs single PUAS)	*2.09 (0.71–6.17)*	*0.18*		
Previous major UAS	0.92 (0.34–2.5)	0.88		
Time period (vs 1997–2008)				
2009–2020	2.02 (0.74–5.5)	0.17		
Spleen-preserving procedure	0.51 (0.17–1.54)	0.23		
Staff surgeon	1.36 (0.56–3.29)	0.49		
Intraoperative unfavorable incident	1.61 (0.48–5.41)	0.44		
Estimated blood loss	1.001 (1.0–1.002)	0.14		
Red blood cell transfusion	1.61 (0.48–5.41)	0.44		
Operative time	1.003 (0.99–1.01)	0.2		

*ASA* American Society of Anesthesiologists; *COPD* chronic obstructive pulmonary disease; *UAS* upper abdominal surgery

Severe complications were observed in 18 patients with open PUAS. Preoperative data, character of complications, and their management are presented in Table 4. The most common indication for LDP among these patients was neuroendocrine neoplasia (7/18 cases). The majority of patients had a history of either major PUAS or nephrectomy (13/18 cases). Half of these patients experienced ≥ 2 complications. POPF or intraabdominal abscess requiring percutaneous drainage/puncture developed in 13 patients. Reoperations were performed for bleeding (*n* = 3), evisceration (*n* = 2), transverse colon necrosis, and perforation (*n* = 1).

**Table 4:** Description of patients with a history of previous upper abdominal surgery experiencing severe complications after laparoscopic distal pancreatectomy

N	Diagnosis	Previous open UAS	Comorbidities	Complications	Outcome
1	PNEN	Cholecystectomy	COPD	Intraabdominal abscess	Percutaneous drainage, AB
2	PNEN	Nephrectomy	-	Evisceration, POPF	Reoperation; percutaneous drainage, AB
3	MCN	Cholecystectomy	-	Pleural effusion; POPF	Percutaneous drainage, AB
4	MTS from RCC	Nephrectomy	RCC; lung MTS	POPF and local abscess	CT-guided puncture, AB
5	PNEN	Nephrectomy	RCC and prostate cancer	Transverse colon necrosis and perforation; POPF; surgical site infection	Reoperation; percutaneous drainage, AB
6	MTS from RCC	Nephrectomy; surgery for aorta aneurysm	Cardiovascular; hypertension; pneumonectomy for lung cancer	Myocardial infarction, POPF	ICU management
7	PNEN	Stomach resection for gastrointestinal stromal tumor	History prostatectomy of prostate cancer	Postoperative bleeding (twice); POPF; pleural effusion	Reoperation (twice); percutaneous drainage, AB; chest tube
8	Accessory spleen	Splenectomy	Spherocytosis	POPF	Percutaneous drainage, AB
9	PNEN	Right hemicolectomy	-	Intraabdominal abscess	Percutaneous drainage, AB
10	PDAC	Left hemicolectomy	Cardiovascular	POPF; pulmonary embolism	Percutaneous drainage, AB; anticoagulant
11	PNEN	Gastrectomy; cholecystectomy; small bowel resection	Cardiovascular; COPD	Cardiopulmonary failure; POPF	ICU management
12	PDAC	Surgery for perforated gastric ulcer; ventral hernia repair	Cardiovascular; hypertension; COPD	Atrial fibrillation; evisceration; POPF; pleural effusion	Volume supply; reoperation; percutaneous drainage, AB, pancreatic duct stenting; chest tube insertion
13	PNEN	Left hemicolectomy	Sigmoid colon cancer	Postoperative bleeding	Reoperation
14	PanIN	Diagnostic laparotomy; Nissen fundoplication	COPD	POPF and local abscess	Percutaneous drainage, AB
15	PDAC	Small bowel resection; colectomy	History of lung embolism	POPF and local abscess	Percutaneous drainage, AB
16	PanIN	Cholecystectomy; liver resection; explorative laparotomy; hiatal hernia repair	Diabetes mellitus	POPF and local abscess	CT-guided puncture, AB
17	MTS from melanoma	Nephrectomy; appendectomy; caecum resection	RCC; caecum cancer	Postoperative bleeding	Reoperation
18	MCN	Cholecystectomy; ventral hernia repair (operated twice)	Hypertension; COPD	Cerebrovascular insufficiency; pulmonary embolism	Death

*UAS* upper abdominal surgery; *PNEN* pancreatic neuroendocrine neoplasm; *MCN* mucinous cystic neoplasia; *MTS* metastasis; *RCC* renal cell carcinoma; *PDAC* pancreatic ductal adenocarcinoma; *PanIN* pancreatic intraepithelial neoplasia; *COPD* chronic obstructive pulmonary disease; *POPF* postoperative pancreatic fistula; *AB* antibiotics; *CT* computed tomography

## Discussion

This is the first study addressing the role of PUAS in the outcomes of LDP. Our findings show that minimally invasive PUAS neither jeopardizes the intraoperative course nor increases postoperative morbidity, mortality, or length of hospital stay following LDP. At the same time, an increased burden and severity of postoperative complications was revealed for open PUAS. This is in contrast to the previous reports on laparoscopic liver, intestinal, and colorectal resections.^10^,^25^–^27^ However, severe complications can hardly be attributed solely to open PUAS. In fact, half of these patients had complications typical for distal pancreatectomy, such as POPF, intraabdominal abscess, and postoperative bleeding. Of note, the rates of POPF and postoperative bleeding were similar between open, minimally invasive and no PUAS. Interestingly, open PUAS was associated with the development of ≥ 2 complications. The latter were present in half of the patients with open PUAS experiencing severe complications. All of these were elderly patients with a history of major PUAS and/or preexisting medical conditions. Thus, this subgroup seems to be the most affected by the negative impact of open PUAS.

Patients with minimally invasive PUAS had perioperative results similar to those of no PUAS. Since the proportion of major PUAS and other preoperative data were comparable between the minimally invasive and open groups, one may conclude that the former has a potential to mitigate the aforementioned risks of open PUAS. This hypothesis has been successfully tested in laparoscopic liver surgery.^28^ However, given the relatively small number of our patients with minimally invasive PUAS and potential risk for type II error, this assumption seems premature. Registry-based cohort studies are needed to evaluate the impact of minimally invasive PUAS on subsequent LDP.

Another important finding of this audit is that LDP following open or minimally invasive PUAS is associated with intraoperative parameters comparable to those in patients without PUAS. Even previous major surgical procedures such as pancreatoduodenectomy did not increase the intraoperative risks of subsequent LDP.^29^ Remarkably, open PUAS did not increase the likelihood of conversion. Data from a high-volume center in the USA suggest that adhesions are one of the most common causes of conversion during LDP.^30^ Unfortunately, the presence of adhesions was not documented routinely in this study. Furthermore, PUAS itself does not necessarily entail extensive adhesions in the abdomen. However, since nearly half of the open PUAS were major, one should expect to have postoperative adhesions in these patients. At the same time, the vast majority (88%) of LDP were performed by senior consultants, thus the risk of conversion could have been diminished by the experience of operating surgeons. Several reports have previously underscored the roles of individual surgeon-volume and institution experience in conversion during minimally invasive distal pancreatectomy.^30^,^31^

The most important drawback of this report is its retrospective design with all inherent biases. Another important limitation is the generalizability of our findings as these are based mostly on a large experience of highly skilled HPB surgeons in an expert center. Finally, the presence of intraabdominal adhesions was not documented routinely, thus there was no possibility to include this parameter in the analysis.

## Conclusion

PUAS itself does not impair the feasibility and safety of LDP as perioperative outcomes are largely comparable to those in patients without PUAS. Open PUAS seems to increase the severity and burden of complications after LDP. This may especially concern the elderly patients with preexisting medical conditions. Further studies should evaluate whether minimally invasive PUAS may mitigate these risks.
